# Liver and spleen elastography of dogs affected by brachycephalic obstructive airway syndrome and its correlation with clinical biomarkers

**DOI:** 10.1038/s41598-020-73209-7

**Published:** 2020-09-30

**Authors:** Andréia Coutinho Facin, Ricardo Andres Ramirez Uscategui, Marjury Cristina Maronezi, Letícia Pavan, Mareliza Possa Menezes, Gabriel Luiz Montanhim, Aparecido Antonio Camacho, Marcus Antônio Rossi Feliciano, Paola Castro Moraes

**Affiliations:** 1 Department of Clinic and Veterinary Surgery, São Paulo State University (UNESP), School of Agricultural and Veterinarian Sciences, Jaboticabal, São Paulo 14884-900 Brazil; 2 Institute of Agrarian Sciences, Federal University of the Jequitinhonha and Mucuri Valleys (UFVJM), Unaí, Minas Gerais 38610-000 Brazil; 3 Department of Farm Animals, Federal University of Santa Maria (UFSM), Santa Maria, Rio Grande do Sul 97105-900 Brazil

**Keywords:** Diagnostic markers, Risk factors, Hypoxia, Respiratory tract diseases

## Abstract

The purpose of this study is to determine whether the brachycephalic obstructive airway syndrome (BOAS) is correlated to alterations in liver and spleen elasticity. Forty-eight brachycephalic and 22 mesocephalic dogs were submitted to a BOAS functional assessment, laboratory tests, abdominal ultrasound and liver and spleen Acoustic Radiation Force Impulse (ARFI) elastography. Dogs clinically affected by BOAS had higher values of liver stiffness (p < 0.001) than healthy dogs: medial lobes (1.57 ± 0.37 m/s), left and right lateral lobes (1.54 ± 0.50 m/s, 1.23 ± 0.28 m/s, respectively) and caudate lobe (1.28 ± 0.42 m/s). Compared to the mesocephalic group, the brachycephalic group (BOAS clinically affected and unaffected dogs) had higher spleen (2.51 ± 0.45 m/s; p < 0.001) and liver stiffness (p < 0.001): medial lobes (1.53 ± 0.37 m/s), left and right lateral lobes (1.47 ± 0.47 m/s, 1.20 ± 0.30 m/s, respectively) and caudate lobe (1.23 ± 0.40 m/s). Principal component analysis explained 70% of the variances composed by liver stiffness increase, erythrocytes and alanine aminotransferase reduction. Brachycephalic dogs had higher spleen and liver stiffness and a subacute inflammatory state, which represent another BOAS systemic effect. Consequently, these dogs can be at higher risk of hepatic disorders compared with mesocephalic dogs, similarly to humans affected by sleep apnea syndrome.

## Introduction

A lately rise in brachycephalic dogs population^1^ has lifted certain concern regarding the welfare and health of these patients^2,3^. These dogs are affected by an upper airway obstruction due to anatomical features intrinsic to the brachycephalic conformation, such as elongated and thickened soft palate, nostril stenosis, tracheal hypoplasia and, moreover, secondary lesions that contribute towards the airway obstruction, with eversion of laryngeal saccules and laryngeal collapse as important factors^4,5^. Named as brachycephalic obstructive airway syndrome (BOAS), this syndrome is recognized as similar to the obstructive sleep apnea (OSA) in humans, and brachycephalic dogs are described as OSA scientific models due to the apnea episodes awake and during sleep^6^.

Significant lower levels of arterial hemoglobin saturation by oxygen was reported in BOAS affected dogs^7,8^ and represent a feature of hypoxemia in these dogs. Upon this precept, patients affected by these syndromes suffer from an intermittent chronic hypoxemia that leads to inflammatory and metabolic dysfunctions^6,8–10^. Among these complications, a liver injury has a critical role in OSA patients^11–14^, as a direct result of liver hypoxia^11^ and metabolic or inflammatory derangements^9,15–17^. The liver injury observed in OSA patients is described as non-alcoholic fatty liver disease (NAFLD) that has a high mortality rate^18–21^ and culminates in liver fibrosis, which is the most important prognostic factor related to NAFLD^19^. The liver injury and fibrosis are also associated with concomitant portal hypertension^20^ that can lead to spleen alterations^21,22^ and changes in serum biomarkers^23,24^.

In this scenario, ultrasound and elastography techniques have been used to diagnose and evaluate liver disorders in both humans^21,22,25–31^ and animals^32–36^. Normal values of liver and spleen stiffness in healthy meso and dolichocephalic dogs measured by Acoustic Radiation Force Impulse (ARFI) elastography are already reported in the literature^37,38^ and recently higher values of liver stiffness were also described in humans affected by severe OSA^30^. However, epidemiologic data regarding hepatic and splenic disorders are absent, as well as if these disorders could be a complication of the intermittent chronic hypoxemia in brachycephalic dogs.

Within this context, the present study hypothesized that the intermittent chronic hypoxemia that affects brachycephalic dogs may develop hepatic and splenic alterations, as observed in humans affected by the OSA. In order to test this hypothesis, this clinical prospective study compared liver and spleen ultrasonography characteristics, elastography stiffness, liver serum biomarkers and blood count between brachycephalic and mesocephalic dogs and between BOAS clinically affected and healthy dogs. All of this in an attempt to predict a secondary effect of the chronic intermittent hypoxia and enhance the understanding of this process in brachycephalic dogs, as it is described in humans affected by OSA. Moreover, we aimed to improve the understanding of BOAS systemic effects and consequently the health care of these animals.

## Results

Out of 50 brachycephalic dogs, 31 French bulldogs and 17 pugs met the inclusion criteria of this study and were included as brachycephalic group (n = 48), and 22 beagles were included as mesocephalic group. Between the brachycephalic and mesocephalic groups, the characteristics genre (females 65% and 55%, respectively), age (3.1 ± 1.5 and 3.7 ± 0.7 respectively) and body condition score (BCS; 6.0 ± 1.3 and 5.95 ± 0.7, respectively) were similar (p > 0.050). Among the brachycephalic dogs, 11/48 (23%) were classified as grade 0 of BOAS (clinically unaffected), 13/48 (27%) as grade I, 13/48 (27%) as grade II, and 11/48 (23%) as grade III. All mesocephalic dogs were considered as BOAS clinically unaffected in this functional assessment.

All animals showed normal B-mode abdominal ultrasonography and any coexisting alterations were ruled out. No differences were verified (p > 0.050) between regions of interest (ROIs) in the evaluated areas of each organ studied. The shear wave velocity (SWV) of cranial, medial, and caudal areas of spleen had no significant difference (p = 0.507), therefore, the average of the three areas was used for further analysis (SpleenSWV). Nevertheless, the SWVs of the hepatic lobes were different (p < 0.001), with exception of the left and right medial liver lobes (p = 0.582). Consequently for the subsequent analyses, the mean SWVs of the medial (MLSWV), lateral left (LLSWV), lateral right (LRSWV), and caudate (CLSWV) lobes were used individually. Between genres, the LiverCLSWV (p = 0.040) platelets (p = 0.012) and direct bilirubin (DB; p = 0.046) were higher in females, and all other parameters were similar (p > 0.050).

**Figure 1:**
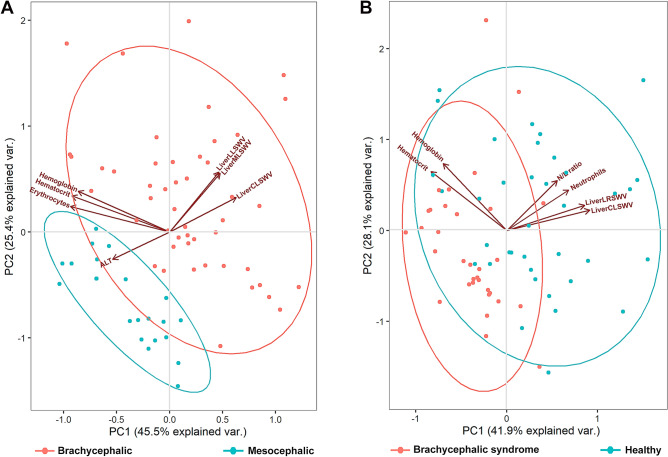
Graphic image of principal component analysis within ARFI elastographic measurements of spleen and liver stiffness, hematologic and serum biomarkers of brachycephalic and mesocephalic dogs (**A**) and of brachycephalic obstructive airway syndrome clinically affected and healthy dogs (**B**). *SWV* shear wave velocity, *LiverLLSWV* left lateral liver lobe, *LiverLRSWV* right lateral liver lobe, *LiverMLSWV* medial liver lobes, *LiverCLSWV* caudate lobe, *N/L* *ratio* neutrophil-to-lymphocyte ratio, *ALT* alanine aminotransferase.

Comparing the skull conformation (Table 1), it was possible to observe that brachycephalic dogs showed higher values of spleen (p = 0.033) and liver lobes (p < 0.001) SWVs, leukocytes (p < 0.001) including neutrophils, lymphocytes, eosinophils and monocytes (p < 0.001), as well as higher value of neutrophil to lymphocyte ratio (N/L ratio; p < 0.001). Mesocephalic dogs had higher values of erythrocytes (p = 0.004), alanine aminotransferase (ALT; p < 0.001), aspartate aminotransferase (AST; p = 0.028) and total bilirubin (TB; p = 0.003). When the variables evaluated were submitted to the principal components analysis, it was possible to observe that 70% of the variability between skull conformations was explained by two components composed by the increase in LiverMLSWV, LiverLLSWV, LiverCLSWV and the reduction of hemoglobin, hematocrit, erythrocytes and ALT (Fig. 1A).

**Table 1:** Mean ± standard deviation of shear wave velocities, obtained by ARFI elastography, values of complete blood count and serum biomarkers between the skull conformations.

	Skull conformation		p-value*
	Brachycephalic	Mesocephalic	
**SWV m/s (± DP)**			
SpleenSWV	2.51 ± 0.45	2.29 ± 0.34	0.033
LiverMLSWV	1.53 ± 0.37	0.96 ± 0.10	< 0.001***
LiverLLSWV	1.47 ± 0.47	0.93 ± 0.11	< 0.001***
LiverLRSWV	1.20 ± 0.30	0.74 ± 0.08	< 0.001***
LiverCLSWV	1.23 ± 0.40	0.76 ± 0.10	< 0.001***
**Complete blood count**			
RBC (μL)	6,930,625 ± 554,879	7,298,636 ± 433,802	0.004**
HGB (g/dL)	17.98 ± 1.63	18.13 ± 1.13	0.670
HCT (%)	50.45 ± 4.54	51.54 ± 3.42	0.271
PLT (μL)	435,708 ± 129,991	387,682 ± 143,212	0.188
WBC (μL)	10,813 ± 2728	8610 ± 1997	< 0.001***
Neutrophil (10^3^/μL)	7715 ± 2385	6058 ± 1456	< 0.001***
Lymphocyte (10^3^/μL)	2155 ± 1016	2007 ± 686	< 0.001***
N/L ratio	4.87 ± 4.00	3.20 ± 0.92	< 0.001***
Basophils (10^3^/μL)	3 ± 15	0 ± 0	0.235
Eosinophils (10^3^/μL)	539 ± 403	241 ± 17	< 0.001***
Monocytes (10^3^/μL)	427 ± 373	367 ± 289	< 0.001***
**Serum biomarkers**			
AST (μL)	28.23 ± 9.74	33.09 ± 7.65	0.028*
ALT (μL)	28.90 ± 11.30	44.40 ± 11.7	< 0.001***
TB (mg/dL)	0.20 ± 0.14	0.31 ± 0.12	0.003**
DB (mg/dL)	0.10 ± 0.11	0.14 ± 0.14	0.245
Total protein (g/dL)	8.54 ± 1.87	7.86 ± 2.40	0.245
Albumin (g/dL)	3.24 ± 0.82	3.44 ± 0.66	0.292

SWV expressed in meters per second (m/s).

*SWV* shear wave velocity, *LiverMLSWV* medial liver lobes, *LiverLLSWV* left lateral liver lobe, *LiverLRSWV* right lateral liver lobe, *LiverCLSWV* caudate lobe, *RBC* red blood cells, *HGB* hemoglobin, *HCT* hematocrit, *PLT* platelet count, *WBC* white blood cells, *N/L ratio* neutrophil-to-lymphocyte ratio, *AST* aspartate aminotransferase, *ALT* alanine aminotransferase, *TB* total bilirubin, *DB* direct bilirubin.

*p-value obtained by covariance analysis (ANOVA). Significance level was set at *p < 0.05, **p < 0.01, ***p < 0.001.

Analysing patients with and without BOAS signs (Table 2), it was possible to observe that BOAS clinically affected patients showed higher values of LiverMLSWV, LiverLLSWV, LiverRLSWV, LiverCLSWV (p < 0.001), leukocytes (p = 0.001) including neutrophils, eosinophils and monocytes (p < 0.001) and N/L ratio (p < 0.001). Healthy dogs had higher values of erythrocytes (p = 0.005), lymphocytes (p < 0.001) and ALT (p = 0.012). When the parameters evaluated were submitted to the principal components analysis, it was possible to observe that 70% of the variability between patients with and without BOAS signs was explained by two components composed by the increase in LiverCLSWV, LiverLRSWV, N/L ratio and neutrophils, beyond reduction of hematocrit and hemoglobin (Fig. 1B).

**Table 2:** Mean ± standard deviation of shear wave velocities, obtained by ARFI elastography, values of complete blood count and serum biomarkers between BOAS affected and healthy dogs.

	BOAS signs		p-value*
	BOAS affected	Healthy	
**SWV m/s (± DP)**			
SpleenSWV	2.47 ± 0.40	2.41 ± 0.45	0.559
LiverMLSWV	1.57 ± 0.37	1.10 ± 0.28	< 0.001***
LiverLLSWV	1.54 ± 0.50	1.03 ± 0.22	< 0.001***
LiverLRSWV	1.23 ± 0.28	0.85 ± 0.23	< 0.001***
LiverCLSWV	1.28 ± 0.42	0.85 ± 0.21	< 0.001***
**Complete blood count**			
RBC (μL)	6,878,919 ± 538,294	7,233,939 ± 494,848	0.005**
HGB (g/dL)	17.82 ± 1.58	18.26 ± 1.33	0.214
HCT (%)	49.94 ± 4.69	51.73 ± 3.47	0.074
PLT (μL)	438,378 ± 123,741	400,697 ± 146,169	0.252
WBC (μL)	11,103 ± 2907	9019 ± 1991	0.001***
Neutrophil (10^3^/μL)	8113 ± 2358	6164 ± 1647	< 0.001***
Lymphocyte (10^3^/μL)	2001 ± 798	2229 ± 1045	< 0.001***
N/L ratio	5.04 ± 3.70	3.56 ± 2.95	< 0.001***
Basophils (10^3^/μL)	1.7 ± 10.35	2.54 ± 14.62	0.295
Eosinophils (10^3^/μL)	554 ± 413	323 ± 280	< 0.001***
Monocytes (10^3^/μL)	466 ± 408	343 ± 255	< 0.001***
**Serum biomarkers**			
AST(μL)	28.27 ± 10.55	31.43 ± 7.64	0.153
ALT (μL)	29.99 ± 12.00	38.03 ± 13.88	0.012*
TB (mg/dL)	0.21 ± 0.15	0.26 ± 0.13	0.161
DB (mg/dL)	0.10 ± 0.12	0.13 ± 0.12	0.298
Total protein (g/dL)	8.60 ± 1.90	8.02 ± 2.21	0.244
Albumin (g/dL)	3.17 ± 0.82	3.46 ± 0.69	0.110

SWV expressed in meters per second (m/s).

*SWV* shear wave velocity, *BOAS* brachycephalic obstructive airway syndrome, *LiverMLSWV* medial liver lobes, *LiverLLSWV* left lateral liver lobe, *LiverLRSWV* right lateral liver lobe, *LiverCLSWV* caudate lobe, *RBC* red blood cells, *HGB* hemoglobin, *HCT* hematocrit, *PLT* platelet count, *WBC* white blood cells, *N/L ratio* neutrophil-to-lymphocyte ratio, *AST* aspartate aminotransferase, *ALT* alanine aminotransferase, *TB* total bilirubin, *DB* direct bilirubin.

*p-value obtained by covariance analysis (ANOVA). Significance level was set at *p < 0.05, **p < 0.01, ***p < 0.001.

**Figure 2:**
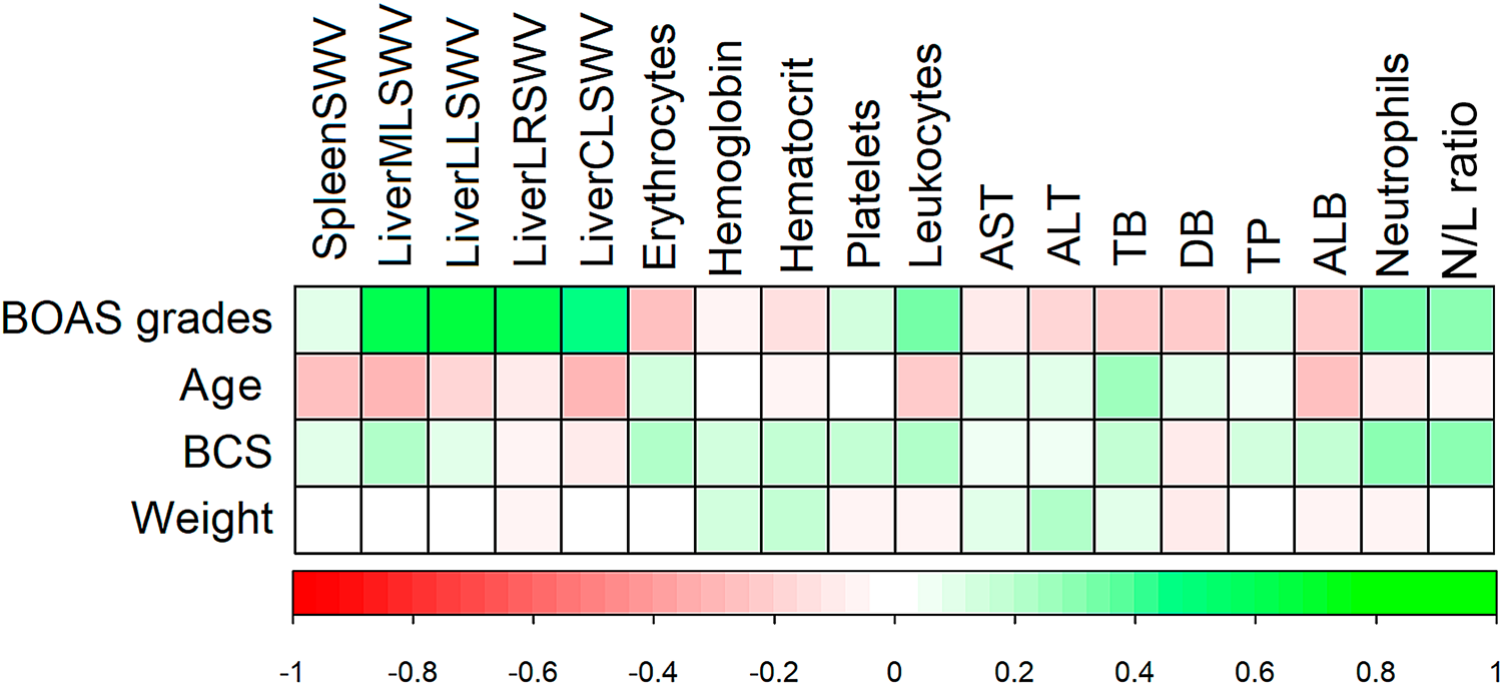
Graphical representation of Spearman's correlation analysis between clinical assessment of brachycephalic obstructive airway syndrome, laboratory and elastographic parameters evaluated in mesocephalic and brachycephalic dogs. *SWV *shear wave velocity, *LiverMLSWV* medial liver lobes, *LiverLLSWV* left lateral liver lobe, *LiverLRSWV* right lateral liver lobe, *LiverCLSWV* caudate lobe, *AST* aspartate aminotransferase, *ALT* alanine aminotransferase, *TB* total bilirubin, *DB* direct bilirubin, *TP* total protein, *ALB* albumin, *N/L ratio* neutrophil-to-lymphocyte ratio, *BOAS* brachycephalic obstructive airway syndrome, *BCS* body condition score.

Evaluating the correlation of these variables (Fig. 2), it was observed that LiverMLSWV (r = 0.555), LiverLRSWV (r = 0.561), LiverLLSWV (r = 0.560), LiverCLSWV (r = 0.315), leukocytes (r = 0.342), neutrophils (r = 0.338) and N/L ratio (r = 0.317) had a positive (p < 0.050) correlation with the BOAS functional grade. LiverMLSWV (r = 0.37), neutrophils (r = 0.319) and N/L ratio (r = 0.289) showed a weak (p < 0.050) correlation with the BCS. Other noteworthy correlation was between SpleenSWV and age (r = − 0.312, p < 0.050). Regarding the correlation between the values of SWV and the depth in which ROIs were defined, the splenic elastography had a minimum depth of 0.70 cm and maximum of 2.8 cm, whilst the minimum and maximum liver depths varied for each lobe: ML 1.2–4.1 cm; LL 1.2–5.9 cm; LR 2.1–6.4 cm; and CL 1.8–7.1 cm. All correlation coefficients were low (< 0.30 or > − 0.30) and without significance (p > 0.05).

## Discussion

Our study showed that brachycephalic dogs had higher values of liver and spleen stiffness when compared to mesocephalic dogs, and BOAS clinically affected dogs had higher values of liver stiffness when compared to healthy dogs. Additionally, brachycephalic dogs had a subclinical inflammatory state evidenced by the neutrophil-to-lymphocyte ratio elevation. Given the lack of data regarding the liver stiffness of brachycephalic dogs our data describe such values, which were also correlated to BOAS degrees and support a possible correlation between BOAS and liver alteration in affected dogs.

The high liver stiffness found in brachycephalic dogs corroborates previously studies in humans affected by OSA^30^, a syndrome directly compared with BOAS, in which the intermittent chronic hypoxia is the most important factor linking this syndrome to liver alterations that conjecture the NAFLD and culminate in fibrosis^13^. Although other studies have considered both grades 0 and I as clinically BOAS unaffected^39^ we have considered only the grade 0 dogs as such and it is noteworthy that we observed the same pattern when comparing this tissue elasticity between dogs clinically affected by BOAS and dogs without clinical signs. Adding to the previously comparison, the positive correlation between BOAS degrees and liver stiffness also suggests that the clinically affected dogs had a worst intermittent chronic hypoxia, which also was previously demonstrated in humans, in which an increase in hypoxemia during sleep was associated with an increased risk of liver disease^30^. Therefore, these data strongly support our hypothesis that the liver alterations observed in the brachycephalic dogs are due to the BOAS intrinsic chronic hypoxemia.

The SWV obtained by elastographic studies depends on the tissue stiffness being highly shaped by fibrosis and has a good predictive value for its presence^22^. Thus, the liver SWV increases along the histopathological grades of liver fibrosis in children and adults^40–42^ as well as in dogs^35,36^. There is no values of liver and spleen stiffness of brachycephalic dogs described, which challenges our data comparison. However, a recent study evaluating liver fibrosis in dogs by shear wave elastography showed that dogs without clinical relevant fibrosis had a median SWV of 1.56 m/s whilst dogs with clinically relevant fibrosis had a median SWV of 2.04 m/s^36^. Although the dogs in the first group were classified without clinically significant liver fibrosis, some were histopathological affected and had similar SWV values to the observed in our brachycephalic dogs. Moreover, our results showed mean liver SWV differences of 0.509 m/s and 0.447 m/s between brachycephalic versus mesocephalic and between BOAS clinically affected versus healthy dogs, respectively. These differences were similar to previous studies that reported mean liver SWV differences of 0.362 m/s between liver fibrosis affected and healthy children^25,40,41,43–45^. Indeed, our study showed even bigger differences between the groups which emphasizes how BOAS can influence this tissue.

Comparatively, the brachycephalic and BOAS clinically affected groups had mean values of liver SWV approximate to the SWV values established as cut-off points for severe fibrosis (1.48 m/s) and cirrhosis (1.63 m/s)^42^. However, it is noteworthy that high measures of liver SWV are described as non-differential between fibrosis and inflammation although evidence shows that higher SWV values were found when liver fibrosis was associated with inflammation in children^41^ and the same pattern was reported in dogs^36^. In this context, the values of liver SWV here obtained on BOAS clinically affected dogs were slightly lower than the cut-off point of 1.7 m/s described to detect liver inflammation or fibrosis in children^41^ but within the range of 1.45–2.24 m/s (median 1.61 m/s) reported in dogs that had hepatic inflammation^36^. We could not define what hepatic alterations were responsible for the higher values of SWV in our brachycephalic dogs, although we suspect that these patients had at least a parenchyma inflammation based on the hematologic results.

The higher values of leucocytes and especially higher N/L ratio, which is a biomarker of subclinical inflammatory response^46,47^, supports the suspicion that these dogs had at least a hepatic parenchyma inflammation. Likewise, humans affected by OSA also demonstrate higher N/L ratio, positively correlated to the OSA severity^48^ and mitigated by continuous positive airway pressure (CPAP) therapy^48,49^. Furthermore, high N/L ratios were reported in dogs affected by systemic inflammatory response syndrome^50^ and septic peritonitis^51^, but not yet described in brachycephalic dogs. Therefore, we considered the existence of a subacute inflammatory status on brachycephalic dogs, which corroborates previously evidence that this syndrome leads to systemic inflammatory derangements^10,52^, and probably to consequences such as observed in humans with OSA.

The chronic intermittent hypoxia induces metabolic abnormalities in humans, which could mediate its effects on the liver and are independent of body mass index^53^. Correspondingly, it could be postulated that the alterations found in our study are a consequence of a metabolic syndrome similarly to the described in the OSA, and the high N/L ratio supports this hypothesis since it is considered as a predictive marker of metabolic syndrome^54^. Unfortunately we did not evaluate others biomarkers of metabolic syndrome in this study, nonetheless, our results supports the existence of a possible metabolic derangement associated to an inflammatory status in brachycephalic dogs affected by the airway obstruction, as previously discussed^10,52^.

It could also be argued that the relationship between the liver stiffness and BOAS might had been affected by body weight and BCS, since visceral obesity was described as an independent predictor of liver fibrosis in NAFLD^55^. However, our results showed that only LiverMLSWV, neutrophils, and N/L ratio showed a weak correlation to the BCS. This weak correlation corroborates a previous study in which severe OSA hypoxemia in patients affected by metabolic comorbidities was correlated with an increased risk of liver disease and liver fibrosis even after body mass index adjustment^30^. Thus, our results could suggest that this phenomenon is also independent of BCS in BOAS affected dogs.

Although the spleen SWV only had a significant difference between the skull conformations in our dogs, this could represent another important factor. Elevated values of spleen SWV were considered as reliable predictor of liver fibrosis^31^ and children affected by liver cystic fibrosis had a mean spleen SWV of 2.51 m/s whilst healthy children had a mean spleen SWV of 2.16 m/s^25^. These findings are similar to our results in which spleen SWV of brachycephalic and mesocephalic groups were 2.51 m/s and 2.29 m/s, respectively. However, a spleen involvement concomitant to the liver injury, induced by the BOAS hypoxemia, cannot be discarded based on our results, given that grade 0 dogs could still show features of airway obstruction by other objective assessments methods as observed previously^39^. The spleen stiffness values for the mesocephalic group were similar to a previously study conducted by our group in mix breed dogs with a similar weight range^37^. The values of liver stiffness of the mesocephalic group here described were lower than the already reported by ARFI technique^38^. However, the latter used a mean SWV for all liver lobes, which could explain this difference. In addition, another study in healthy beagles had a median SWV of 1.51 m/s^56^ but these values were obtained only from the right lateral lobe in eight dogs.

The mesocephalic group had higher levels of ALT, AST and TB. ALT and AST are used to evaluate liver necroinflammatory lesions in dogs and can also have higher activity due to other sources, as well as the total bilirubin used to evaluate liver function^57^, which could explain our findings. In contrast, patients affected by severe OSA had different levels of liver serum biomarkers^11^ and elastography studies has shown excellent correlation between the SWV values and biochemical predictors of liver fibrosis (ratio AST/ALT) and between levels of liver biomarkers individually (ALT, AST, GGT and total bilirubin)^30,58^. Whereas, the ratio AST/ALT has no significant correlation to liver fibrosis in dogs and actually has an overlap of values between healthy and affected dogs^24^ and, therefore, the ALT and AST enzymatic activities were evaluated individually in this study.

As our results showed, the brachycephalic dogs had an indicative of a subacute inflammation state. Although the design of this study was cross-sectional, it would not be possible to determine whether BOAS preceded the onset of increased liver stiffness. However, the anatomical components that cause BOAS are congenital^59^ and thereby we can assume that this syndrome, and its intrinsic hypoxemia, preceded indeed the liver alterations observed in our patients. Thus, strengthen the link between BOAS and liver alterations. Whether the N/L ratio and liver alterations could be useful as a prognosis basis of BOAS remains unclear and further studies are required.

It is important to emphasize that the components that cause the airway obstruction in brachycephalic dogs are, in its majority, liable of surgical correction. Thus, the surgical treatment of such components could lead to a decrease of the chronic intermittent hypoxia that affect these dogs, similarly to the CPAP therapy implemented in OSA human patients. Both treatments focus on reduction of hypoxia, which could therefore diminish its systemic effects and metabolic or inflammatory derangements. Clearly, our results reassures the need of surgical correction of BOAS components in an early age as previously recommended^59^.

Finally, the comparison of SWV values between studies is limited by factors linked to the elastography technique itself. A variety of elastographic techniques are available and could be divided between strain and shear wave imaging^60^, which should be interpreted carefully when comparing different studies. The ARFI quantification technique used here is a method of shear wave imaging in which the measurements are not a result of the compression force applied by the operator^60^, hence these values are less prone to be affected by the operator, with good agreement for intra and inter-operator^61^. Moreover, the use of ARFI quantification SWV facilitates the comparison between our results and other shear wave imaging studies, as previously discussed.

Another important point of discussion is the anatomical location where the quantitative measurements were obtained. Human studies have used the left or right lateral liver lobes for quantitative elastographic evaluations^25,62^ and dogs studies have described SWV values of the right lateral liver lobe^36,56^ although some reports do not define the exactly liver lobe where the measurements were performed^33,38^. In order to have a complete evaluation of the entire parenchyma, as well as to detect possible focal lesions, our methodology included the evaluation of all liver lobes and three splenic regions, thus ensuring a complete evaluation and facilitating the comparison with future studies. Lastly, the depth in which the SWV are measured could affect these quantitative measurements as already reported in children^25^ and dogs^38^, in which the depth had a significantly and negative correlation with the SWV values. Our results did not showed significant correlation between the depths and SWV values, which corroborates others previously studies^43,63^.

Our study had limitations. First, the lack of histopathological assessment limits our results. The risks of life-threatening complications associated to a liver biopsy has an important impact on the research boundaries and acceptance by the dog’s owners. Therefore, our results provide a basis and justification to further histopathological investigation that are needed to determine which pathological process could affect the liver in BOAS affected dogs, sensitivity, specificity and a cut-off value of ARFI elastography for distinguishing such lesions. Moreover, specific measurements of portal blood flow would be of interest to ensure if the changes of spleen stiffness in brachycephalic dogs observed could reflect portal hypertension. However, the brachycephalic dogs without any sedation did not tolerate the time needed to perform such measurements and factors such as tachypnea in these dogs affected directly the reliability of such measurements. Secondly, we could not find any associated change in the liver enzymes measurements here described and did not performed others biomarkers such as bile acids that were not available for our study at the time. This may have limited the understanding of the process here investigated and furthers studies are required to investigate whether the increased liver and spleen stiffness are due only to the hypoxemia or if there is any other factors involved, as well as its impact on liver function. Finally, we like to emphasize the learning curve over the course of the study regarding the functional assessment of BOAS and its effect could have biased the assessment of BOAS grades in some dogs.

In conclusion, brachycephalic dogs, specifically pugs and French bulldogs, showed higher spleen and liver stiffness when compared with mesocephalic dogs. In the absence of histopathological scores in our study, further studies are required to stablish which specific lesion these dogs could have, prove the spleen involvement and stablish cut-off points to detect a possible liver fibrosis, inflammation or fatty liver disease due to the BOAS hypoxemia as reported in the OSA scenario. Our study has proven that BOAS could have one more consequence due to its hypoxia and the presence of a subacute inflammation state in brachycephalic dogs. These findings reassure the similarity between BOAS and OSA. The ARFI elastography technique represents a helpful and non-invasive tool to detect liver and spleen alterations in brachycephalic dogs and it might be useful as a prognosis tool. Finally, this study underlines the risk of liver lesions in brachycephalic dogs, especially liver inflammation or fibrosis, due to BOAS systemic effects.

## Methods

### Ethical aspects

This study followed the recommendations of the Brazilian National Council for the Control of Animal Experimentation (CONCEA) and was approved by the Institutional Ethics Committee in the Use of Animals of the São Paulo State University (Unesp), School of Agricultural and Veterinarian Sciences, Jaboticabal, São Paulo, Brazil (protocol no. 17944/17). The owners of dogs selected for this study signed an informed consent for their animal’s participation.

### Study design and animals

This was a prospective, observational, case–control study performed between April 2018 and July 2019. Fifty brachycephalic dogs, pugs and French bulldogs, from partner breeders and owners were selected and composed the brachycephalic group. Twenty-two beagle dogs, from the institutional animal nutrition laboratory, were selected and comprised the mesocephalic group, according to the following defined criteria. The sample size was calculated based on previously results^64^ and 15 mesocephalic and 30 brachycephalic dogs were needed to allow identification of a minimum 0.4 m/s differences on the shear wave velocity of the hepatic parenchyma, with a statistical power of 89%, using a 95% significance level. The final selection of 48 brachycephalic dogs and 22 mesocephalic allowed an increase of the statistical power to 95%.

As inclusion criteria, it was defined that the animals had to belong to the aforementioned breeds, be adults between 1 to 6 years old, body weight between 5 to 15 kg and present an updated control of infectious diseases. The following exclusion criteria were defined: dogs that underwent any previously surgical procedures for relieve of BOAS signs, or that presented clinical signs of systemic diseases in the time of the evaluation and/or in the previous six months and aggressive animals that do not allow the proposed evaluations.

### Clinical assessment

All procedures were applied in the Veterinary School Hospital “Governador Laudo Natel” of São Paulo State University Campus of Jaboticabal installations. Initially, clinical examination was carried out on all patients, to determine their health status and BCS. Subsequently, blood samples were collected to complete blood count and measurement of serum biomarkers, ALT, AST, TB, DB, total protein and albumin. The animals were then taken to the ultrasound laboratory, where B-mode ultrasound and elastography were performed, as detailed below.

Once the ultrasound examination was completed, the animals were submitted to the BOAS functional assessment, performed in a 3-min exercise tolerance test followed by laryngeal auscultation as described and validated by Riggs et al. (2019)^39^. This test classified dogs accordingly to the severity of pre and post-exercise clinical signs, into the BOAS grades 0 to 3 in which grade 0 is considered as non-affected and grade 3 as severe affected. Only one trained evaluator (ACF) conducted the test. After the trial period, the animals had a follow up of 2 months to ensure health status.

### Ultrasound assessment

**Figure 3:**
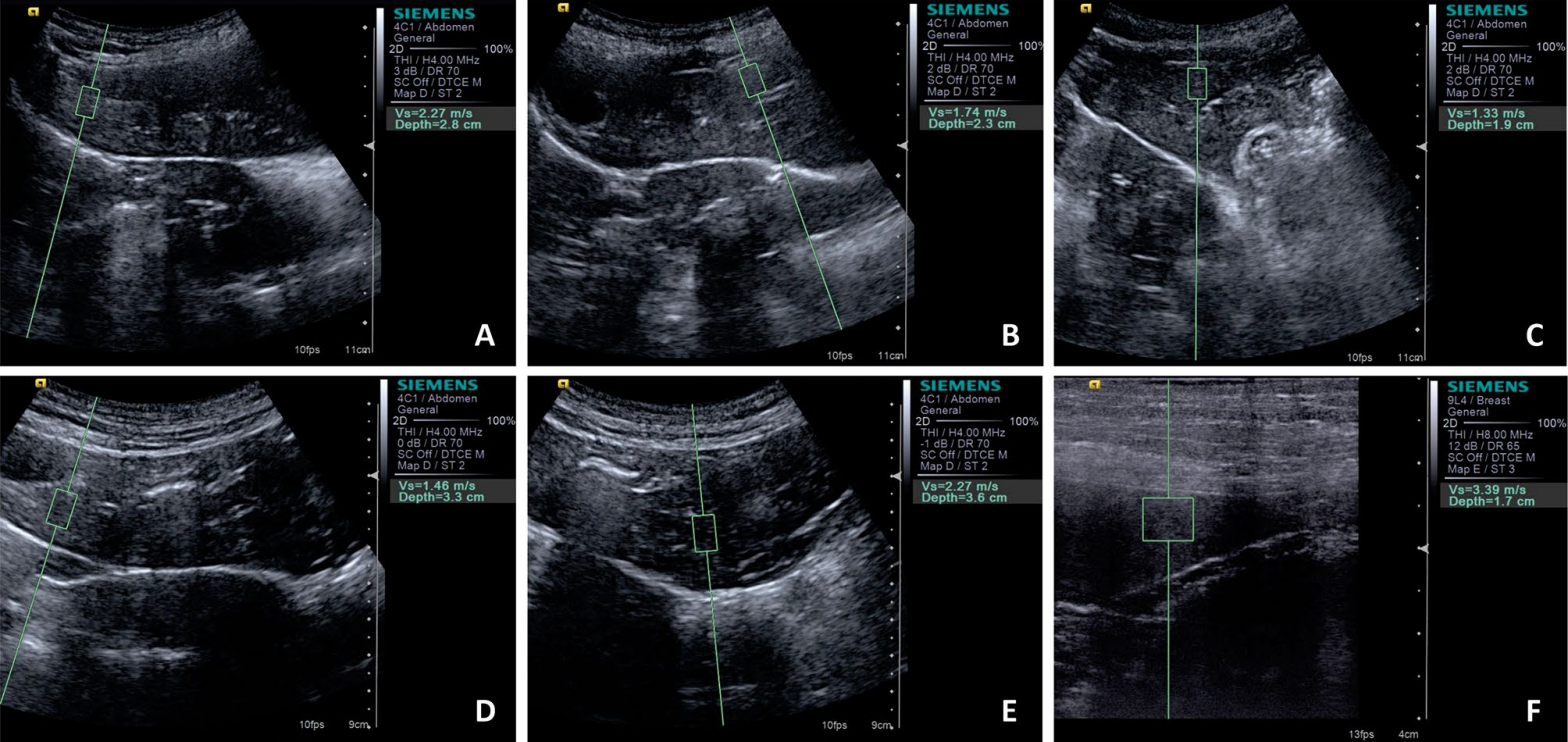
Quantitative ARFI-elastography images of the liver lobes and spleen of a brachycephalic dog. (**A**) Right medial, (**B**) left medial, (**C**) left lateral, (**D**) right lateral lobe, (**E**) caudate lobe and (**F**) spleen.

To allow a complete evaluation of all liver lobes and splenic parenchyma according to the required ultrasound window, the animals were positioned in dorsal, right lateral and left lateral recumbency. The hair of the abdominal area was amply clipped to facilitate transcutaneous ultrasonographic examinations and hydrophilic gel was applied to the abdominal skin and the surface of the probe to ensure appropriate contact. No sedation was required throughout the entire scanning. The routine abdominal ultrasound examination was performed after 12 h of solid fasting by a single experienced sonographer (radiologist with 8 years of experience; MSc. PhD. and Post-Doctoral Degree; MCM) and coexisting alterations were ruled out. Hepatic and splenic parenchyma were located and evaluated using the Siemens Acuson S2000 ultrasonic device (Munich, Germany) equipped with a convex (4 MHz) and linear (9 MHz) transducers. Subsequently the ARFI elastographic ultrasound software (Virtual Touch Tissue Quantification; Siemens, Germany) was activated, getting an ultrasound image B-mode, qualitative elastogram (grey scale in which darker areas represent soft and lighter hard tissues). For elastographic quantitative evaluation, at least three ROI were selected by placing the 5 × 5 mm pre-defined calliper over the areas evaluated: left and right medial liver lobes, left and right lateral liver lobes, caudate liver lobe; and in three spleen regions, caudal, medial and cranial (Fig. 3). The ultrasound software automatically provides the SWV (m/s) for each of these ROI. The choice regarding the performance of quantitative measurements in all liver lobes and three splenic regions was made in order to provide a thorough evaluation of the entire parenchyma.

### Statistical analysis

Statistical analysis was performed with help of R software version 3.3.0 (R Foundation for Statistical Computing, Austria). First, the Shapiro–Wilk Bartlett tests were used to validate normality distribution and homoscedasticity of the variances, respectively, of all studied variables. Subsequently, the variation between the ROIs of each tissues area and the variation between the areas were evaluated using the Bland–Altman concordance analysis test. The measurements resulting from clinical, laboratory and ultrasound analyses were then compared between the skull conformation (brachycephalic and mesocephalic dogs) and between BOAS clinically affected and non-affected (healthy) dogs using the *t* Student test. The variables that presented significant differences were submitted to a principal component analysis in order to identify which components could explain the variability between brachycephalic and mesocephalic dogs and between BOAS affected and non-affected dogs. Finally, the correlation between the parameters defined by the clinical assessment and the variables studied was analysed by the Spearman test. The significance was fixed in 95% (p < 0.05) for all tests.

## Data Availability

The datasets generated and analysed during the current study are available from the corresponding author on reasonable request.
